# Supplementary material for: Different environmental gradients affect different measures of snake β-diversity in the Amazon rainforests
Source: PeerJ. 2018 Sep 24;6:e5628. doi: 10.7717/peerj.5628 (PMC6162079; doi:10.7717/peerj.5628)
Supplement: Supplemental Information 1 [file peerj-06-5628-s001.doc]

**SUPPLEMENTARY INFORMATION**

**Table S1.** Snake species found in RAPELD sampling modules distributed along 880 km of tropical rainforest in the Brazilian Amazonia. The numbers of modules can be used to check for geographic location of modules in the map shown in Figure 1. The “X” symbol means species occurrence in a module

| **Taxon** | **Modules where each species was found (see Fig. 1)** |
| --- | --- |
| **Boidae** |  |
| *Boa constrictor* | 1, 4 |
| *Corallus hortulanus* | 5, 8, 9, 12, 13, 14, 15, 17, 18, 19, 20 |
| *Epicrates cenchria* | 9, 12, 16, 19 |
| **Colubridae** |  |
| *Chironius fuscus* | 1, 3, 4, 5 |
| *Drymoluber dichrous* | 1, 2, 3 |
| *Rhinobothryum lentiginosum* | 18, 19 |
| *Spilotes pullatus* | 8, 13 |
| *Taeniophallus brevirostris* | 1, 2 |
| **Dipsadidae** |  |
| *Atractus schach* | 6 |
| *Clelia clelia* | 1, 2, 5, 13, 14 |
| *Dipsas indica* | 12, 18, 20 |
| *Drepanoides anomalus* | 2, 5, 6, 11, 16, 17, 19, 20 |
| *Erythrolamprus reginae* | 21 |
| *Erythrolamprus typhlus* | 1, 12 |
| *Helicops angulatus* | 8, 10, 11, 14 |
| *Imantodes cenchoa* | 1, 2, 3, 4, 5, 6, 7, 8, 9, 10, 14, 15, 18, 19, 21 |
| *Imantodes lentiferus* | 10 |
| *Leptodeira annulata* | 1, 5, 6, 9, 12, 16, 18, 19, 21 |
| *Oxyrhopus occipitalis* | 9, 19 |
| *Oxyrhopus melanogenys* | 5, 6, 7, 9, 11, 13, 16, 18, 21 |
| *Philodryas argentea* | 1, 16, 17, 20 |
| *Pseudoboa coronata* | 19,18 |
| *Siphlophis compressus* | 1, 2, 3, 12, 14, 16, 17, 19, 20 |
| *Xenopholis scalaris* | 7, 8, 19 |
| **Viperidae** |  |
| *Bothrops atrox* | 1, 2, 3, 4, 5, 7, 8, 9, 11, 14, 15, 16, 17, 18, 19, 20, 21 |
| *Bothrops taeniatus* | 8, 9 |

**Table S2.** Biological traits used to estimate functional diversity of 26 snake species from the Amazon rainforests. Continuous traits were measured in five specimens per species and we used average values per species. Discrete traits were obtained in the literature and complemented with field observations.

| **Trait** | **Relevance** | **References** |
| --- | --- | --- |
| Total length | May differ among areas because of local genetic variability and the effects of prey availability on growth rates. Additionally, populations of snakes in the Amazon may be structured by associations between body size and environmental gradients such as distance from drainage. | Berry et al., 1987  Dobson and Murie, 1987  Ebenhard, 1990  Madsen and Shine, 2013  Fraga et al., 2013 |
| Tail length | May be an important factor affecting dispersal capacity along heterogeneous habitats, because it defines snakes ability to cross patches of different habitats. Snake assemblages may be different among different habitats because of variation in tail length. | Guyer and Donnelly, 1990  Martins et al., 2001  Cavalheri et al., 2015 |
| Eye diameter | Varies according to habitat use and period of activity. Large eyes seem to improve ability to visually avoid diurnal predators. | Bonnet et al., 1999  Liu et al., 2012 |
| Maximum size of offspring | Affects populations structure by determining the number of individuals that reaches maturity. Therefore, offspring size may be related to variation in density and geographic range. Additionally, it may be mediated by energy intake, specially in viviparous. | Ford et al., 1989  Seigel and Ford, 1992  King, 1993 |
| Discrete habitat | It is directly resulted from the environmental heterogeneity. High availability of different habitats is expected to generate greater diversity of species with different lifestyles in an assemblage. Additionally, habitat selection has physiological consequences, which influences mortality. | Vitt and Vangilder, 1983  Webb et al., 2004  Bernarde and Abe, 2006 |
| Period of activity | Defines interactions with prey and predators, which implies in morphological and behavioral adaptations to survival and locomotor performance. Additionally, drives hunting and resting sites, which has implications to physiological factors such as thermoregulation. | Moore, 1978  Webb and Shine, 1998  Webb et al., 2004  Llewelyn et al., 2006 |
| Foraging mode | Affects dispersal capacity and consequently gene flow. Ambush predators may have population structure more affected by geographic and environmental distances than active foragers. Additionally, affects energy intake. | Huey and Pianka, 1981  Secor amd Nagy, 1994  Fraga et al., 2017 |
| Diet | Defines energy intake and foraging frequency. Variation in prey availability across landscapes may generate variation in species co-occurrence and diet plasticity. | Daltry et al., 1996  Santos et al., 2000  Barlow et al., 2009 |
| Defensive behavior | Drives interspecific interactions, which is directly associated to population stability through suitable balance between birth and mortality rates. Additionally, it may be temperature-dependent. | Scudder and Burghardt, 1983  Herzog et al., 1989  Keogh et al., 1994 |
| Reproductive mode | Determines the amount of energy necessary to reproduction, and the behavior of pregnant females. | Shine, 1980  Luiselli et al., 1996  Shine, 2003 |

**Table S3.** Ecological gradients used as independent variables in multiple linear regression models to investigate factor affecting snake diversity in the Amazon.

| **Gradient** | **Amplitude** | **Average** | **Data collecting** | **Justification** |
| --- | --- | --- | --- | --- |
| Geographic distance | 1–871.39 (converted to km) | 357.67 (converted to km) | Coordinates X and Y per sampling module | A complex variation in environmental heterogeneity occurs in the Amazon along the latitudinal and longitudinal axes, which mainly resulted from climate variation and the Andean uplift. Therefore, environmental filtering should be tested independently of geographic distance. |
| Clay content in the soil | 13.08–73.13 | 34.61 | Measured by fieldwork | Soil texture affects the overall ecosystem productivity and causes variation in microclimate. |
| Height Above the Nearest Drainage (HAND) | 2.64–69.64 | 21.4 | Raster surface | Distance from drainage is an important factor structuring animal and plant assemblages in the Amazon, including snakes. |
| Percentage of tree cover | 16–88 | 65.4 | Raster surface | Variation in tree cover causes variation in light intensity, temperature and availability of resting and nesting sites for snakes. |

**Table S4.** Summary of RDA analyses testing the effects of ecological gradients on snake β-diversity measures. CP values show the constrained proportion of variance on diversity measures captured by RDA. Inertia values are equivalent to variance. P-values were estimated by 5000 ANOVA with 5000 randomizations. Bolded P-values indicate cases in which null hypothesis was reject. All the environmental data were converted into residuals from linear regressions applied to reduce the effects of geographic distance on environmental heterogeneity. Clay = clay content in the soil (%). HAND = Height Above the Nearest Drainage.

| Diversity measure | CP | Inertia | Clay | | HAND | | Tree cover | | Temperature | | Precipitation | |
| --- | --- | --- | --- | --- | --- | --- | --- | --- | --- | --- | --- | --- |
|  |  |  | F | P | F | P | F | P | F | P | F | P |
| TBD | 0.32 | 0.32 | 2.59 | **0.01** | 1.71 | 0.12 | 1.38 | 0.24 | 0.57 | 0.76 | 0.84 | 0.55 |
| FBD | 0.43 | 1.85 | 0.21 | 0.82 | 7.51 | **0.002** | 1.35 | 0.24 | 0.60 | 0.52 | 1.70 | 0.23 |
| PBD | 0.36 | 7.64 | 2.07 | 0.08 | 4.08 | **0.005** | 1.10 | 0.31 | 0.43 | 0.87 | 0.89 | 0.49 |


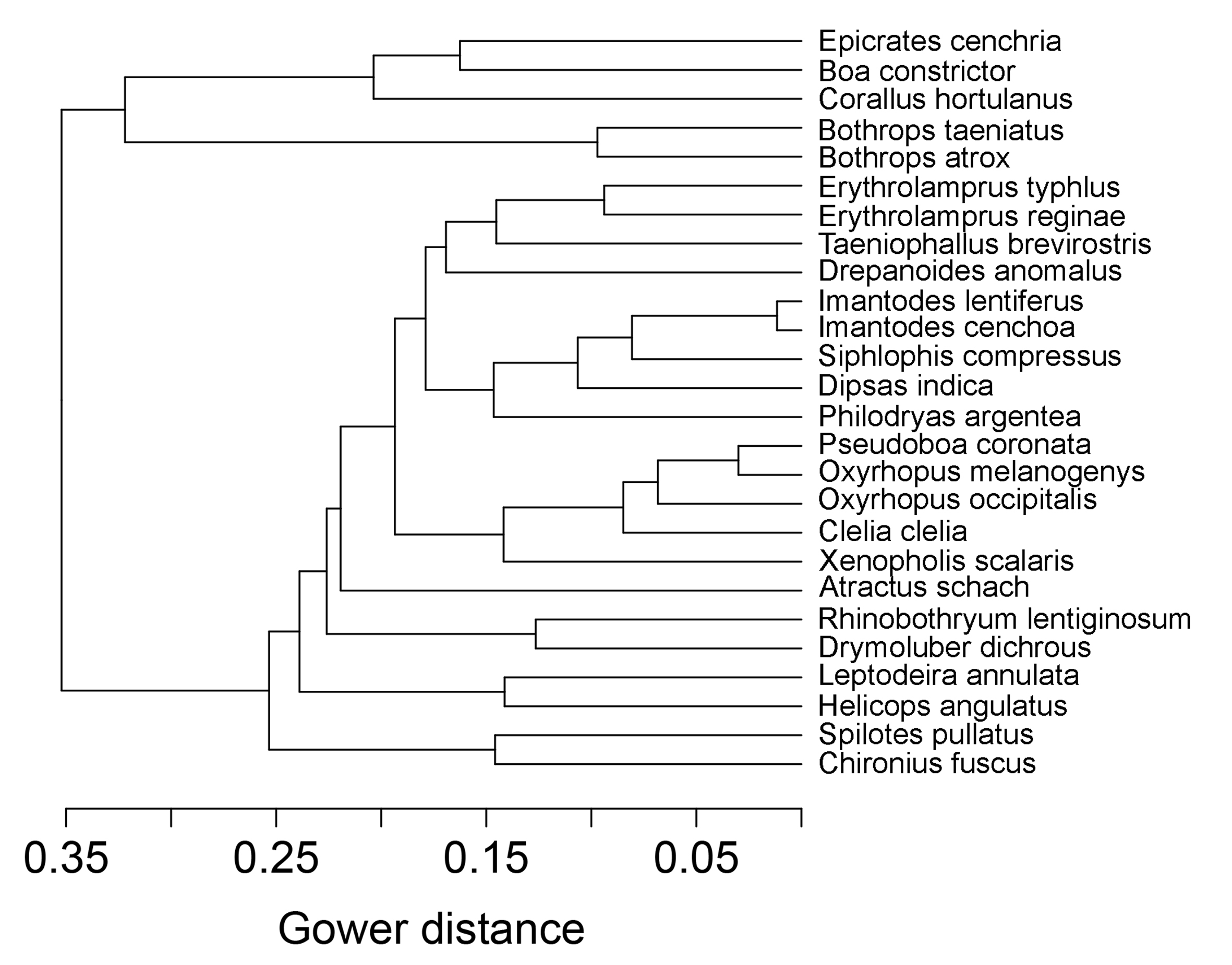
**Fig. S1.** Functional tree of snakes from the Amazon based on UPGMA (underweighted pair-group method with arithmetic mean). Function was estimated by Gower distances among ten continuous and discrete traits per species.

**Fig. S2.** Phylogenetic tree of snakes from Amazonia adapted from Pyron et al (2013). Relationships among species were estimated by maximum-likelihood analysis of 12 genes (5 mtDNA and 7 nuclear) concatenated. Numbers at nodes are support values estimated by non-parametric Shimodaira-Hasegawa-Like implementation of the approximate likelihood-ratio test. Small numbers are branch lengths imported to estimate phylogenetic β-diversity (< 0.04 are omitted).


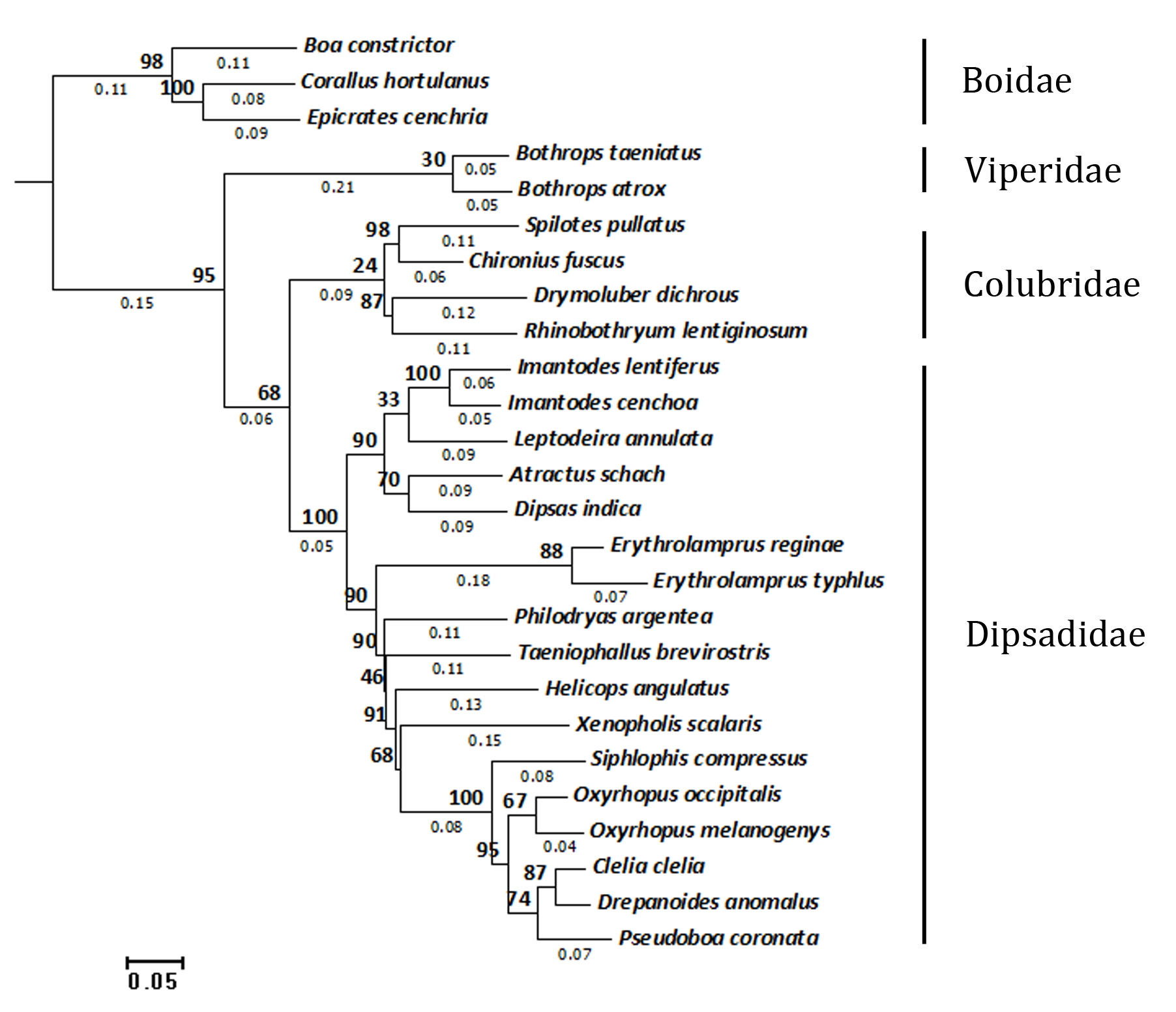


**Fig. S3.** Spatial correlograms implemented in the ecodist R-package (*Goslee & Urban, 2007*) showing spatial autocorrelation in residuals returned by multiple-linear-regressions based on taxonomic (A), functional (B) and phylogenetic (C) β-diversity estimates as dependent variables, and gradients of clay content in the soil, Height Above the Nearest Drainage (HAND), percentage of tree cover, temperature of the coldest month and precipitation of the wettest month as independent variables. Black circles indicate spatial autocorrelation at P < 0.05.

**
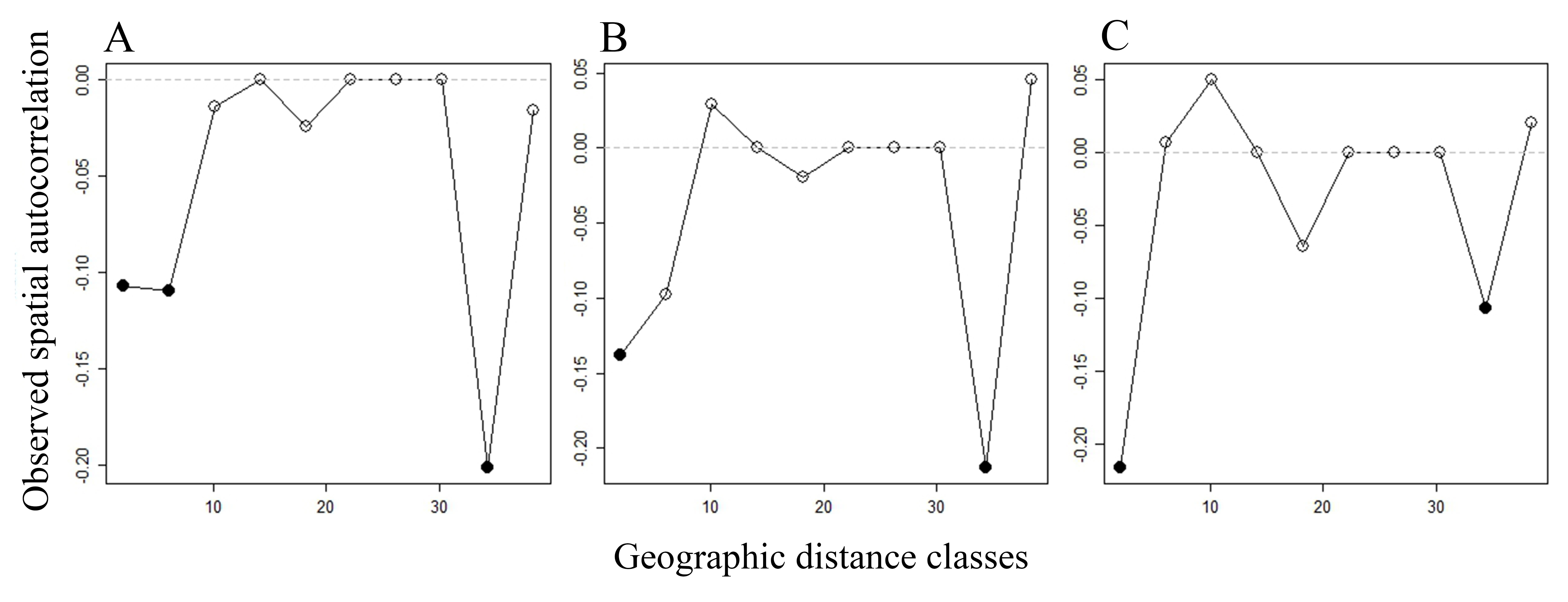
**
